# CFS-1686 Causes Cell Cycle Arrest at Intra-S Phase by Interference of Interaction of Topoisomerase 1 with DNA

**DOI:** 10.1371/journal.pone.0113832

**Published:** 2014-12-02

**Authors:** Ru-Wei Lin, Chia-Ning Yang, ShengYu Ku, Cheng-Jung Ho, Shih-Bo Huang, Min-Chi Yang, Hsin-Wen Chang, Chun-Mao Lin, Jaulang Hwang, Yeh-Long Chen, Cherg-Chyi Tzeng, Chihuei Wang

**Affiliations:** 1 Bone and Joint Research Center, National Cheng Kung University, Tainan, Taiwan; 2 Medical Device R & D Core Laboratory, National Cheng Kung University Medical College and Hospital, Tainan, Taiwan; 3 Department of Life Sciences, National University of Kaohsiung, Kaohsiung, Taiwan; 4 Department of Biotechnology, Kaohsiung Medical University, Kaohsiung, Taiwan; 5 Department of Orthopedics, Kaohsiung Medical University Hospital, Kaohsiung, Taiwan; 6 Department of Biochemistry, School of Medical, Taipei Medical University, Taipei, Taiwan; 7 Department of Medical and Applied Chemistry, Kaohsiung Medical University, Kaohsiung, Taiwan; German Cancer Research Center, Germany

## Abstract

CFS-1686 (chemical name (E)-N-(2-(diethylamino)ethyl)-4-(2-(2-(5-nitrofuran-2-yl)vinyl)quinolin-4-ylamino)benzamide) inhibits cell proliferation and triggers late apoptosis in prostate cancer cell lines. Comparing the effect of CFS-1686 on cell cycle progression with the topoisomerase 1 inhibitor camptothecin revealed that CFS-1686 and camptothecin reduced DNA synthesis in S-phase, resulting in cell cycle arrest at the intra-S phase and G1-S boundary, respectively. The DNA damage in CFS-1686 and camptothecin treated cells was evaluated by the level of ATM phosphorylation, γH2AX, and γH2AX foci, showing that camptothecin was more effective than CFS-1686. However, despite its lower DNA damage capacity, CFS-1686 demonstrated 4-fold higher inhibition of topoisomerase 1 than camptothecin in a DNA relaxation assay. Unlike camptothecin, CFS-1686 demonstrated no activity on topoisomerase 1 in a DNA cleavage assay, but nevertheless it reduced the camptothecin-induced DNA cleavage of topoisomerase 1 in a dose-dependent manner. Our results indicate that CFS-1686 might bind to topoisomerase 1 to inhibit this enzyme from interacting with DNA relaxation activity, unlike campothecin's induction of a topoisomerase 1-DNA cleavage complex. Finally, we used a computer docking strategy to localize the potential binding site of CFS-1686 to topoisomerase 1, further indicating that CFS-1686 might inhibit the binding of Top1 to DNA.

## Introduction

Human topoisomerase type 1 (Top1), a member of the topoisomerase family, is responsible for DNA topological problems associated with supercoiling [1]. Top1 catalyzes single-stranded DNA cleavage and relegation, required to relax DNA supercoiling generated by replication, transcription and chromatin remodeling [2]. Mechanically, this enzyme performs its function by first forming a phosphotyrosine intermediate between the tyrosine of Top1 and the phosphate of the DNA backbone, resulting in a DNA break [3]. Then, the 5′ end of the break strand rotates around the intact strand to unwind the supercoils, and the break strand is religated to free tyrosine [4].

Because of its crucial function for cells, especially in DNA replication, Top1 has become an attractive drug target for anticancer chemotherapy [5]. Several anticancer drugs drive cancer cells toward apoptosis by inducing the Top1-DNA cleavage complex (Top1-DNAcc) [6]. They are roughly classified into two groups, camptothecin (CPT) with CPT derivatives and non-CPT Top1 inhibitors, all belonging to the interfacial inhibitor of Top1-DNAcc [6]. These inhibitors bind reversibly at the interface of Top1-DNAcc to stabilize this transient complex [7]. This action might slow down the Top1 catalytic cycles, leading to DNA damage as the fast movements of the replication complexes collide with this drug-stalled complex. So far, only CPT derivatives such as topotecan and irinotecan have been approved by the FDA as Top1-targeted drugs for various forms of cancer. Several non-CPT Top1 inhibitors are still in clinical development [4]. In addition to the interfacial inhibitors of Top1-DNAcc, the catalytic inhibitors of Top1 might be worth developing for clinical and Top1 mechanistic studies [4]. The compounds in this category can inhibit the DNA relaxation of Top1 and the formation of Top1-DNA complexes, but they cannot induce Top1-DNAcc [8]–[10].

We have identified a series of (*E*)-2-(2-(5-nitrofuran-2-yl)vinyl)quinoline derivatives that effectively induced cell cycle arrest at S phase in both PC3 and LNCaP cells, consequently triggering apoptosis [11]. In the current study, we characterized the most potent compound from this series, CFS-1686, to determine its Top1 activity. We compared the effect of CFS-1686 with CPT on cell cycle progression in PC3 cells by BrdU incorporation and flow cytometry analysis, revealing that CFS-1686 and CPT induce cell cycle arrest at the intra-S phase and G1-S, respectively. Further evaluation of their capacity for DNA damage assessed by the phosphorylation of ATM and by the level of γH2AX and its foci demonstrated that CFS-1686 caused light DNA damage, whereas CPT caused heavy DNA damage. CFS-1686 inhibited Top 1 activity 4-fold more than CPT in a DNA relaxation assay, but nevertheless did not induce DNA cleavage. However, it reduced CPT-induced DNA cleavage of Top1 in a dose-dependent manner, suggesting that CFS-1686 might bind to Top1 to inhibit this enzyme from interacting with DNA. Using a docking strategy, we identified a potential binding site of CFS-1686 in Top1, showing that it might compete with DNA at the DNA binding site of Top1.

## Materials and Methods

### Cell culture and synchronization

PC3 cells were purchased from the Bioresource Collection and Research Center (BCRC) in Taiwan. The cells were seeded at 5×10^5^ cells/per plate (10 cm) in RPMI 1640 with 10% fetal bovine serum. For synchronization, thymidine was added to 2 mM after 12 hr and incubated for another 16 hours. The cells were released by washing three times with PBS and re-fed with fresh serum-rich medium for 8 hours. Then cells were re-fed with fresh media containing 2 mM thymidine for 16 hours. The cells were washed by PBS three times before subsequent steps.

### BrdU incorporation assay

About 5×10^3^ cells/per well were seeded into 96-well plates. After 12 hr, the cells were incubated with 1 µM of CPT (Sigma), 1 µM of CFS-1686 or DMSO as a control. BrdU incorporation assays were performed using the BrdU cell proliferation kit (Roche). BrdU labelling and detection followed the manufacturer's protocol. Briefly, cells were pulsed with BrdU for 5 hr and then fixed and denatured, followed by immunodetection of BrdU incorporation Absorbance was measured at 370 nm.

### Flow cytometric cell cycle analysis

The synchronized PC3 cells were incubated with 0.5 µM of CPT, 0.5 µM of CFS-1686 and DMSO as a control, respectively. The cells were harvested by trypsinization after compound treatments of 0 and 5 hr, centrifuged, washed with PBS and collected by centrifugation. The cells were fixed with ice-cold 70% ethanol for 30 min, washed with PBS and centrifuged to remove supernatant. The cells were re-suspended in PBS containing 0.05% Triton X-100 and RNAase A (40 µg/mL) and incubated at 37°C for 1 hr, and propidium iodide (PI) was added to the cell suspension to a final concentration of 50 µg/mL for another 1 hr incubation. The cells were harvested by centrifugation, washed with PBS and centrifuged to remove supernatant. Finally, the cells were re-suspended in PBS and analyzed by flow cytometer (BD Biosciences) with Software (BD Biosciences).

### Total cell lysate preparations and immunoblotting

The synchronized PC3 cells were incubated with 0.5 µM of CPT (Sigma), 0.5 µM of CFS-1686, and DMSO, respectively, for 0, 1, 2, 3, 4, and 5 hr. Then the cells were subjected to sonification and centrifuged to remove cell debris, and the supernatants were collected. Protein concentration was determined by a protein assay kit (PIERCE). About 40 µg of protein/per well was resolved in electrophoresis aparatus. After electrophoresis, the proteins were transferred to a nitrocellulose membrane. The transferred membranes were blocked in 5% (w/v) nonfat dry milk in TBST (0.5 M NaCl, 20 mM Tris-HCl, 0.05% (v/v) Tween 20, pH 7.4) and probed for antibody against ATM (Cell Signaling), Phospho-ATM (Ser-1981; Cell Signaling), γH2AX (Cell Signaling), PARP, caspase 3 activation form and GAPDH (Cell Signaling), followed by incubation with a secondary antibody conjugated horseradish peroxidase (anti-rabbit, anti-mouse, anti-goat; Jackson ImmunoResearch) and visualization with chemoluminiscence substrate (Millipore) using the chemoluminiscence detection system (BIO-RAD) or exposing to X-ray film.

### Immunofluorescence analysis with a confocal microscope

5×10^4^ PC3 cells were plated on sterile 18-mm glass coverslips. Cells were cultured with CFS-1686 or CPT for 12 hours in the presence of CPT or CFS-1686, fixed in methanol (-20°C, 10 min) and perforated with 0.1% triton ×100 (25°C, 2 min). Cells were blocked in 3% bovine serum albumin-TBS and then stained with primary γH2AX monoclonal antibody followed by secondary antibodies with fluorophores 488 nm or 555 nm (Invitrogen). The coverslips were mounted with anti-fade reagent with DAPI (Invitrogen) upside down on glass slides. The images were obtained using confocal microscope (ZEISS) and amplified 63 and 150 times.

### Recombinant human TopI protein expression and purification

The production of human Top1 proteins was carried out using a baculoviral expression system in Sf-9 insect cells. The detailed methods for protein production and purification were described previously [12].

### DNA relaxation assay

The inhibitory effect of CPT and CFS-1686 on supercoiled DNA strand breakage caused by TopI was evaluated. Plasmid pUC19 DNA (200 ng) was incubated at 37°C for 30 min in a reaction solution (40 mM Tris-acetate, 100 mM NaCl, 2.5 mM MgCl2, and 0.1 mM EDTA; pH 7.5) in the presence or absence of different concentrations of inhibitors in a final volume of 20 µl. The conversion of the covalently closed circular double-stranded supercoiled DNA to a relaxed form was used to evaluate DNA strand breakage induced by TopI. Samples were loaded onto a 1% agarose gel, and electrophoresis was performed in TAE buffer (40 mM Tris-acetate and 1 mM EDTA). The gel was stained with ethidium bromide (0.5 µg/mL) for 5 min, then photographed under transmitted ultraviolet light.

### DNA cleavage assay

Plasmid pUC18 DNA was digested by Hind *III* to expose the 5′ overhang end. Then [γ-32P]dATP was added onto its 5′-end by T4 polynucleotide kinase (New England Biolabs). Labeled DNA was diluted to a concentration of 100,000 C.P.M to achieve its best activity. [γ-32P] labeled pUC18 was incubated with human recombinant topoisomerase I and various concentrations of CFS-1686 or CPT in reaction buffer (10 mM Tris-HCl (pH 7.5), 50 mM KCl, 5 mM MgCl2, 0.1 mM EDTA and 15 mg/ml BSA) for 20 min at room temperature. The reactions were stopped by adding 10% SDS to a final concentration of 5%, and two volumes of loading dye (80% (vol/vol) formamide, 10 mM NaOH, 1 mM EDTA, 0.25% (wt/vol) bromophenol blue and 0.25% (wt/vol) xylene cyanol) were added. Before loading into wells, the reactions were heated at 95°C for 5 min to denature recombinant topoisomerase I. Then aliquots of every reaction were loaded into 6% natural gel (12 ml 40% acrylamide gel, 12 ml 5× TBE, 300 µl APS, 20 µl TEMED in 36 ml DDH_2_O), and run at constant voltage of 300 V for 6 hours. Afterward, gels were dried at 80°C for 60 min and then exposed to imaging plate (FUJIFILM) and scanned by imaging systems (FUJIFILM).

### Molecular docking

A docking study was performed using a LigandFit module in the Discovery Studio 2.1 version (Accelrys). The structure of the top1 protein has been solved only in the closed form, when it is fully embracing the DNA, and no information is available concerning its possible conformation in the absence of the substrate. The X-ray structure of top1 in complex with DNA was taken (PDB entry code: 1K4S) [13] and refined as follows. The bound DNA substrate was removed to generate a pseudo open state of top1 and the phosphorylated Tyr723 was modified to a nonphosphorylated state. The modified protein structure was subjected to minimization using Steepest Descent and Conjugate Gradient methods with backbone constraint to allow side chains to find their proper orientations. Previously, the structure of top1 without DNA substrate was predicted by molecular dynamics simulations [14] where an X-ray structure of human top1 with the elimination of 22-bp DNA duplex oligonucleotide was used as a starting configuration. The modeling result indicated that the protein undergoes a large conformational change due to linkage between core sub-domains I (a.a. 215-232 and 320-433) and III (a.a. 434-635) which opens up leaving the rearrangement in the orientation of the protein domains, while their secondary and tertiary structures are maintained. Accordingly, the DNA contact surface of the pseudo open state of top1 generated in our approach is proper for docking simulation. The structure of CFS1686 was then drawn into the Discovery Studio package with a CHARMm force field and minimized using Steepest Descent and Conjugate Gradient methods. The docking scoring function used in this study considers two energy terms, the internal energy of the ligand and the interaction energy of the ligand with the receptor. A higher positive score means a better docking behavior.

## Results

### CFS-1686 and CPT have different effects on cell cycle progression

**Figure 1:**
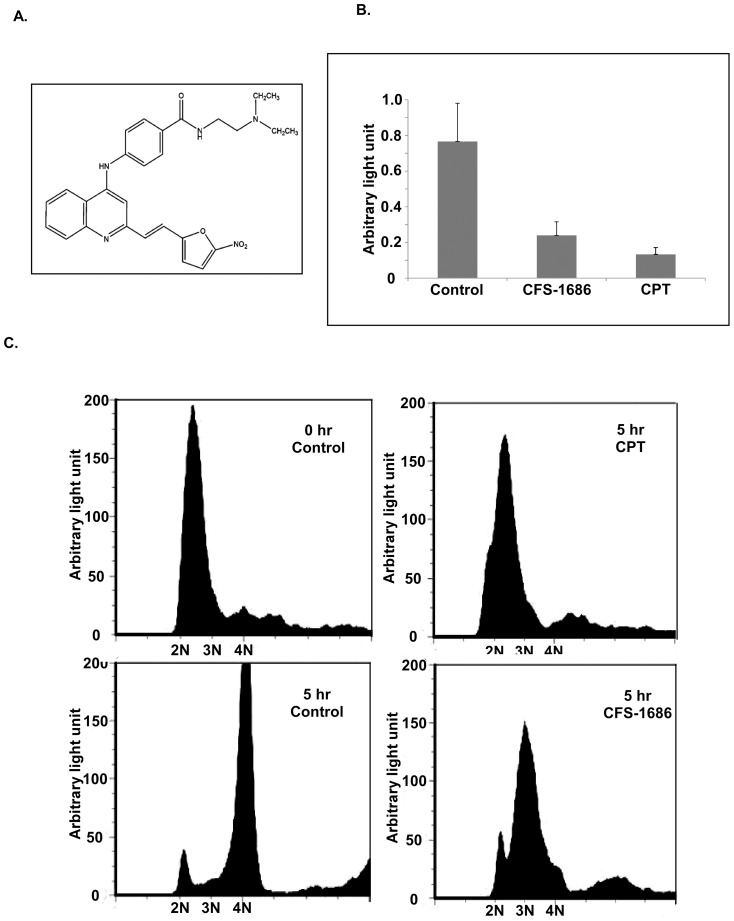
CFS-1686 and CPT induced cell cycle arrest at the G1/S boundary and intra-S in PC3 cells, respectively. (A) The chemical structure of CFS-1686. (B) BrdU incorporation of synchronized PC3 cells treated by CFS-1686 or CPT for over 5 hrs. (C) Flow cytometric analyses of synchronized PC3 cells treated by CFS-1686 or CPT for over 5 hrs. Analyses were in triplicate and representative histograms are shown. The X and Y axes represent DNA content and cell numbers, respectively.

Our previous study identified a series of (*E*)-2-(2-(5-nitrofuran-2-yl)vinyl)quinoline derivatives that showed cytotoxic effects on both PC3 and LNCaP cells [11]. Among the compounds from this series, CFS-1686 demonstrated the highest potency against PC3 cell proliferation (figure 1A). To decipher the mechanism by which this compound affects cells, we first compared the effect of CFS-1686 with CPT on cell cycle progression. By using a BrdU incorporation assay to measure the efficiency of DNA replication, we observed that CFS-1686 and CPT reduced DNA synthesis by about 70% and 80%, respectively, in comparison with control cells treated with DMSO (figure 1B). Furthermore, cytometric analysis showed that cells treated by CPT remained at the G1-S boundary, whereas the cells treated by CFS-1686 entered into the stage between G1 and G2, mainly at intra S-phase (figure 1C).

### CFS-1686 led to a late checkpoint response for DNA double-strand break in comparison with CPT

**Figure 2:**
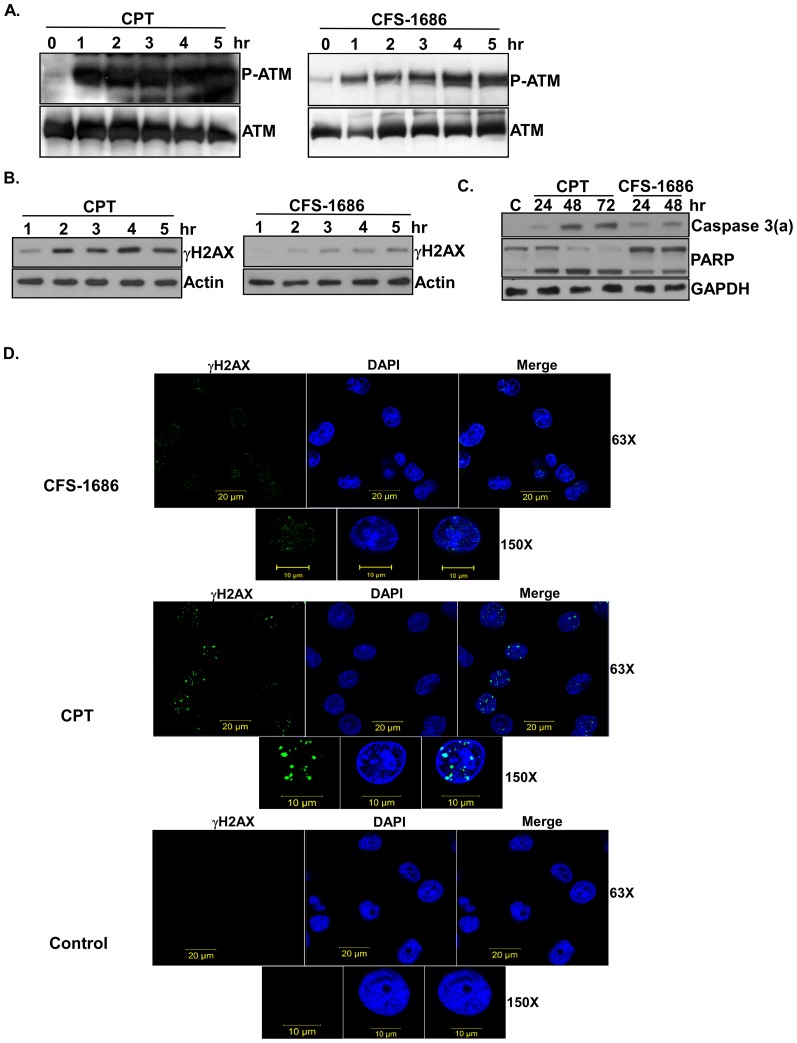
The effects of CFS-1686 or CPT on DNA damage and apoptosis in PC3 cells. (A) Phosphorylation of ATM at Ser1981 was induced by CFS-1686 or CPT. (B) Increased levels of γH2AX were induced by CFS-1686 or CPT. (C) CFS-1686 or CPT could activate caspase 3 to cleave PARP. (D) γH2AX foci appeared in cells treated with CFS-1686 or CPT. For immunoblot analysis, cell lysates from PC3 cells treated by CFS-1686 or CPT were assayed over the indicated time courses to detect the phosphorylation of ATM at Ser1981 and γH2AX, the activation of caspase 3 and the degradation of PARP. P-ATM stands for the phosphorylation form of ATM. Caspase 3(a) represents the activation form of caspase 3. Experiments were repeated twice and representative results are shown. For immunofluorescence micrographs of γH2AX, PC3 cells treated by CFS-1686 or CPT for 12 hours were fixed and stained with the antibody for γH2AX and DAPI. Analyses were duplicated and representative immunofluorescence micrographs are shown. γH2AX was shown by green color. DNA was shown by blue color. 63X: 63 fold amplification in the magnitude. 150X: 150 fold amplification in the magnitude.

We investigated whether CFS-1686-induced cell cycle arrest at the intra-S phase was due to DNA damage. The master regulators of DNA damage are ATM and ATR [15]. Since CPT, by causing a DNA double strand break, activates ATM by auto-phosphorylation on Ser1981 to initiate cell cycle arrest and DNA repair [16], we asked if CFS-1686 or CPT could increase the level of ATM auto-phosphorylation. Western blotting analysis with a specific antibody against P-Ser1981 of ATM demonstrated that ATM auto-phosphorylation resulted and reached saturation within 1 hr after CPT treatment, whereas auto-phosphorylation in cells treated by CFS-1686 was slight after 1 hr and progressed toward saturation after 4 hr (figure 2A).

We then examined the levels of DNA double-strand breakage in CPT- and CFS-1686-treated cells using an antibody against γH2AX, which is the target of ATM and a DNA double-strand break marker [17]. As expected, γH2AX appeared and became saturated after 2 hrs of CPT treatment (figure 2B), right after CPT-induced ATM activation (figure 2A). In contrast, γH2AX started to appear after 2 hr and reached saturation after 4 hr in CFS-1686-treated cells (figure 2B), also correlating with the timing of ATM phosphorylation (figure 2A). We further examined the formation of γH2AX foci in the cells treated with CPT or CFS-1686 and demonstrated that CFS-1686 induces many light and small spots mixed with DNA while CPT induces the obvious γH2AX foci mixed with DNA (figure 2D). These results suggested that CPT might cause much higher degree of DNA damage than that of CFS-1686.

Apparently, both CPT and CFS cause DNA damage, resulting in activation of ATM to trigger DNA damage repair. The main differences between CFS and CPT were in the timing of the DNA damage and the levels of damage. We speculated that CFS-1686 might, like CPT, interact with Top1, but in a different way, with different results for this DNA topology enzyme.

### CFS-1686 induced apoptosis in PC3 cells

We next asked if CFS-1686-induced DNA damage can lead cells toward apoptosis, by measuring the activation of caspase 3 and the degradation of PARP. Clearly, like CPT, CFS-1686 could efficiently activate caspase 3 to further cleavage of PARP (figure 2C). Interestingly, the degree of cellular apoptosis induced by CFS-1686 and CPT exactly matched their respective capacity for DNA damage.

### CFS-1686 inhibited Top1 activity in DNA relaxation assays but had no activity in DNA cleavage assays

**Figure 3:**
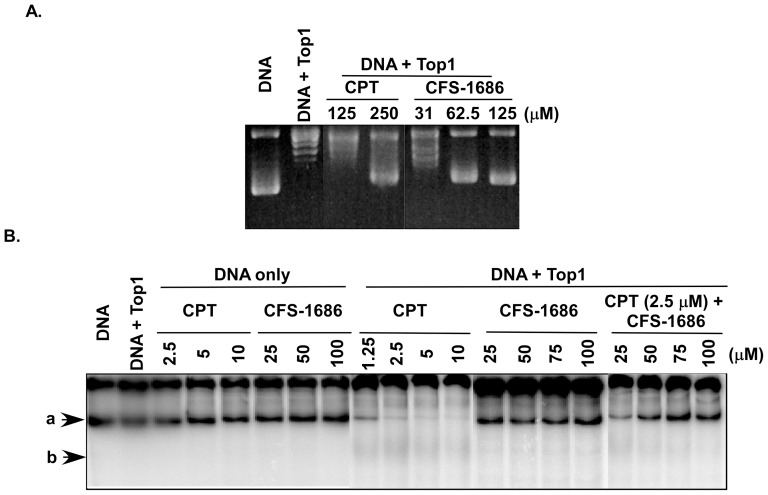
The effects of CFS-1686 or CPT on Top1-mediated DNA relaxation and cleavage. (A) CFS-1686 demonstrated 4-fold higher inhibition of Top1-mediated DNA relaxation than CPT. Recombinant Top1 enzymes were incubated with plasmid pUC19 DNA with/without CFS-1686 or CPT. Agarose electrophoresis was run to resolve the supercoiling or relaxed form of DNA. (B) CFS-1686 could not induce Top1-DNAcc, but it reduced CPT-induced DNA cleavage of Top1. The recombinant Top1 enzymes were incubated with γP^32^-labelled pUC19 DNA with/without CFS-1686 or CPT. Then natural PAGE was run to resolve the intact and cleavage products. Experiments were repeated twice and representative results are shown. Arrows “a” and “b” indicate the compacted form of DNA and smear cleavage DNA, respectively.

We performed DNA relaxation assays to ask if CFS-1686 affects Top1 to relax the supercoiling form of DNA. As shown in figure 3A, we demonstrated that CFS-1686 had high potency to inhibit Top1 DNA relaxation activity. CPT could inhibit Top1 DNA relaxation activity at around 250 µM, but we did not observe any effect at 125 µM (figure 3A). In contrast, CFS-1686 caused inhibition at concentrations as low as 62.5 µM, about 4-fold more potent than CPT (figure 3A).

We then used a modified DNA cleavage assay to investigate whether CFS-1686 interacted with Top1 to inhibit religation of Top1-mediated DNA cleavage. The results clearly demonstrated that CPT inhibited religation of DNA cleavage at concentrations as low as 12.5 µM, resulting in the disappearance of the long length compacted DNA (figure 3B). CFS-1686 had totally no effect on this action, but nevertheless it reduced CPT-induced DNA cleavage in dose-dependent manner (figure 3B). We speculated that CFS-1686 might inhibit Top1 activity by a different mechanism than CPT to affect the interaction of Top1 with DNA and preclude formation of the Top1-DNA complex.

### CFS-1686 could dock into the DNA binding site of Top1

**Figure 4:**
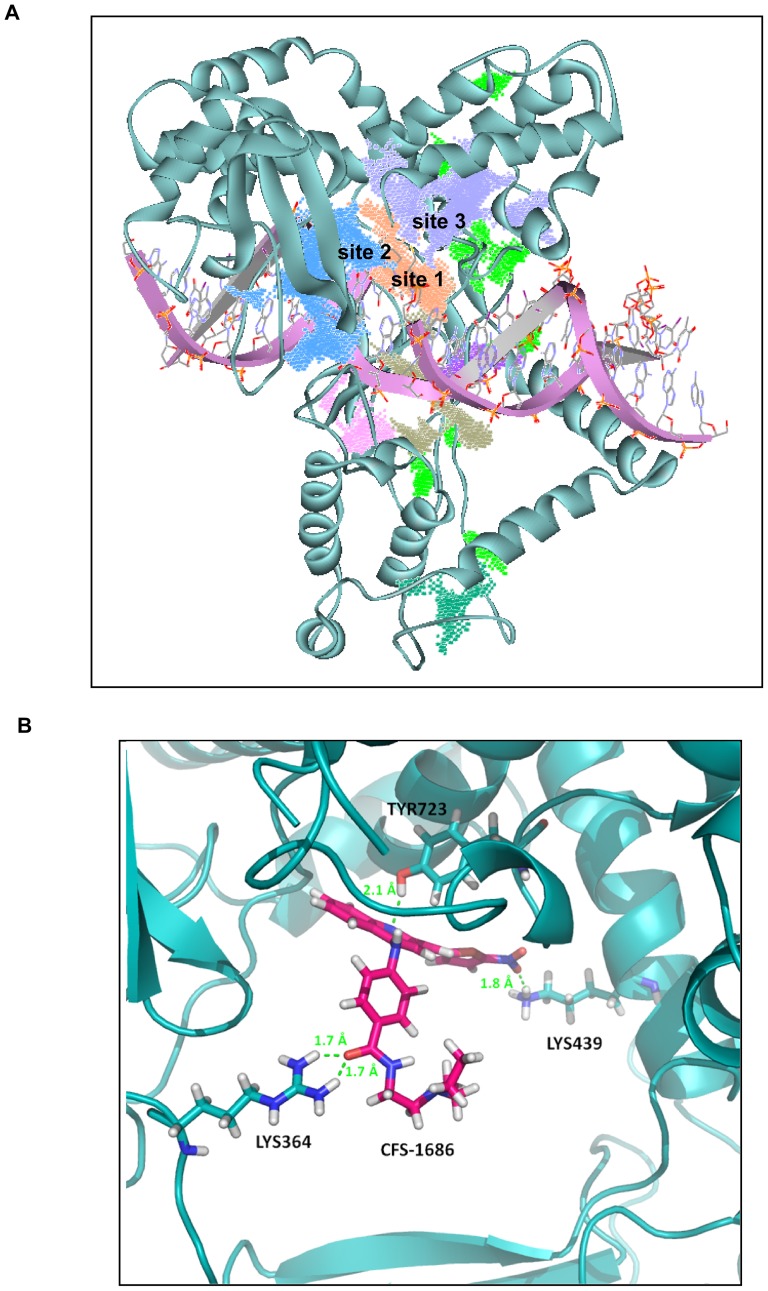
CFS-1686 could dock into the DNA binding site of Top1. (A) Three possible docking sites of CFS-1686, cited as 1, 2 and 3, were found in the interface of the top1-DNA complex. Site 1 was the best choice for docking simulation. (B) Detailed interaction of CFS-1686 at site 1. A LigandFit module was used to find the docking sites of CFS-1686 at the interface of the Top1-DNA complex.

Our results indicated that CFS-1686 might interact with top1 prior to the top1-DNA complex formation. We localized the interaction sites of Top1 and CFS-1686 by first defining several possible docking sites in the interface of top1 and DNA substrate. The LigandFit module located several possible docking sites in this region (figure 4A). Except for sites 1 to 3, most docking sites were too shallow to accommodate the compounds and therefore they were excluded. Sites 2 and 3 were so bulky that when the DNA substrate was superposed back to the pseudo open state of Top1, a compound docked at these sites might not interfere with the incoming DNA substrate. Consequently, site 1 was the best choice for the docking simulation. Site 1 is also adjacent to Tyr723, whose phosphorylation is greatly involved in cleavage of the 3′-phosphotyrosine ester bond of the DNA substrate [18], [19]. This further supports our choice of docking site.

The detailed interaction of CFS-1686 at site 1 is shown in Figure 4B. The positively charged guanidinio side chain of Arg364 anchors the CFS1686 carbonyl oxygen atom through hydrogen bonding, and the nitro group points toward top1 Lys439 for a hydrogen bond formation. Meanwhile, an additional hydrogen bond is formed between the nitrogen atom of the CFS1686 quinoline group and the Tyr723 hydroxyl oxygen atom. The docking score of CFS1686 is 74. Altogether, it is the nitro group that can interact with both Lys443 and Lys587 simultaneously or merely Lys439 as an essential structural element to maintain the studied compound's potency at this docking site. To examine this necessity, we docked compounds 5, 6, 7, 8, and 16 [11] to this site. Their docking scores as well as that of CFS1686 are listed in Table 1. Compounds 5, 6, 7, and 8 were drawn into the package and prepared as CFS-1686. As expected, compounds 5, 6, 7, and 8, which lack a nitro group, have lower docking scores ranging from 32 to 40, whereas both 16 and CFS-1686 carrying a nitro group have better scores, 62 and 74 respectively.

**Table 1:** Docking score of CFS-1686 in comparison with five studied compounds (11).

Compound	Dock score
CFS-1686	74
Cpd16	62
Cpd05	32
Cpd06	38
Cpd07	32
Cpd08	40

## Discussion

Our previous study identified a series of compounds with cytotoxic effects on prostate cancer cell lines [11]. Among them, CFS-1686 showed the highest capacity for inhibiting cell proliferation. The current study demonstrated that this compound has a different mechanism from CPT to induced cell cycle arrest at intra-S phase, resulting in late and light DNA damage. It showed 4-fold higher activity for the inhibition of Top1 than CPT in DNA relaxation assays. Unlike CPT, CFS-1686 had no activity on Top1 in DNA cleavage assays, but nevertheless it reduced CPT-induced DNA cleavage of Top1 in a dose-dependent manner. We concluded that CFS-1686 might affect the interaction of Top1 with DNA to preclude formation of Top1-DNA complexes. This speculation was further supported by computer docking simulations showing a potential CFS-1686 binding site in the Top1-DNA interacting interface.

**Figure 5:**
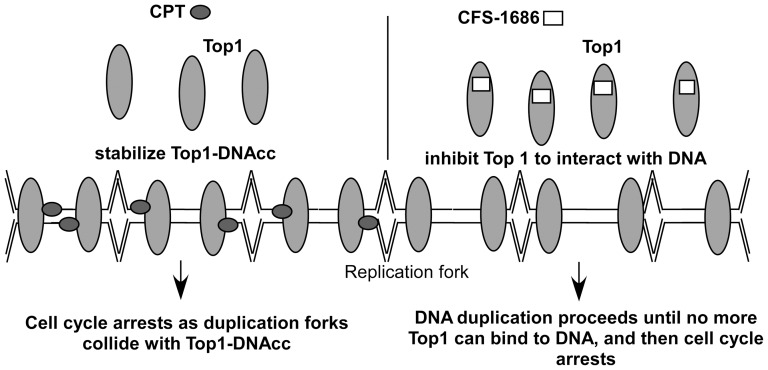
Proposed interaction mode of CPT or CFS with Top1 during DNA replication.

The most interesting issue raised by the current study is why CFS-1686 could cause such different outcomes from CPT in cell cycle arrest and DNA damage. To address that, we first considered DNA duplication after thymine-induced synchronization. The synchronized PC3 cells stayed mainly at G1-S phase (figure 1C). DNA replication might already start at this stage (figure 5). Reasonably, many Top1 molecules might already sit on the DNA to perform its cleavage and re-ligation for relaxing DNA supercoiling generated by replication (figure 5). As CPT intercalates into Top1-DNAcc to stabilize this complex, the replication fork will collide with it to generate a DNA double-strand break, resulting in the maximum checkpoint response within 2 hr (figure 2). Instead of acting on Top1-DNA cc, CFS-1686 might affect the interaction of Top1 with DNA to inhibit Top1-DNA binding, eventually reducing Top1-mediated DNA relaxation (figure 5). We hypothesized that DNA replication can go forward at the early stage after CFS-1686 exposure, until the replication machinery requests Top1 to relax DNA supercoiling (figure 5). Without enough Top1 to relax the DNA supercoil form, the stress generated by DNA replication might cause DNA damage and activate the cell cycle checkpoint (figure 5). Thus, the cell cycle arrest induced by CFS-1686 appeared later, and the magnitude of DNA damage was lighter than that of CPT (figure 2).

Another interesting issue is the modified DNA cleavage assay in the current study. Unlike the pilot method [20], we used 3000 bp of linear DNA as a substrate of Top1 instead of 180 bp of short DNA, and used 6% natural PAGE instead of denatured sequencing gel to run electrophoresis. Using natural PAGE resolved DNA into a slow and fast migration band, which represented a relaxed and a compacted form of DNA, respectively (figure 3B). CPT-induced DNA cleavage only occurred in the compacted DNA (figure 3B), reflecting that Top1 only acts on the compacted DNA and not on the relaxed DNA. In addition, we did not observe any specific sizes among the cleavage products of DNA other than the smear and the faint band on the gel (figure 3B), which is different from previous findings [21]. This result suggested no sequence preference for CPT to interact with Top1-DNAcc for the long length supercoil DNA.

In summary, the current study has identified a novel catalytic inhibitor of Top1, CFS-1686, which can induce cell cycle arrest at the intra-S phase and cause DNA damage in PC3 cells. Our docking strategy further deciphered its mode of action by direct contact with essential amino acids of Top1, including Lys364, Lys439 and Tyr723 located at the interface of the DNA-Top1 interaction, to inhibit relaxation of Top1 upon DNA replication.
